# Detection of macrolide and fluoroquinolone resistance-associated 23S rRNA and parC mutations in Mycoplasma genitalium by nested real-time PCR

**DOI:** 10.3389/fcimb.2023.1271392

**Published:** 2023-10-20

**Authors:** Wenyin He, Ying Yuan, Junyu Liang, Xuejiao Fan, Lei Li, Xingfei Pan

**Affiliations:** ^1^ Department of Clinical Laboratory, Guangdong Provincial Key Laboratory of Major Obstetric Diseases, Guangdong Provincial Clinical Research Center for Obstetrics and Gynecology, The Third Affiliated Hospital of Guangzhou Medical University, Guangzhou, China; ^2^ Department of Infectious Diseases, Guangdong Provincial Key Laboratory of Major Obstetric Diseases, Guangdong Provincial Clinical Research Center for Obstetrics and Gynecology, The Third Affiliated Hospital of Guangzhou Medical University, Guangzhou, China

**Keywords:** *Mycoplasma genitalium*, macrolide, fluroquinolone, 23S rRNA, parC, nested real-time PCR

## Abstract

**Background:**

Traditional drug susceptibility testing cannot be performed in clinical laboratories due to the slow-growing characteristics of *Mycoplasma genitalium* when cultured *in vitro*. Sanger sequencing is the standard method for detecting drug resistance-associated mutations. It has been used in some laboratories to guide the choice of macrolide antibiotics for *Mycoplasma genitalium* infected patients. Furthermore, resistance to fluoroquinolone has become another emerging clinical challenge.

**Objective:**

Sequencing analysis can detect unknown mutations, but it is time-consuming, requires professional analytical skills and the appropriate testing equipment. The main objective of this study was to establish a nested real-time PCR method for the simultaneous detection of *23S rRNA* and *parC* genotypes in relation to the macrolide and fluoroquinolone resistance.

**Results:**

105 MG-positive samples and 27 samples containing other pathogens were used for validation. The limit of the nested real-time PCR detection was 500 copies/reaction and there was no cross-reaction with *Ureaplasma urealyticum*, *Mycoplasma hominis*, *Chlamydia trachomatis*, *Neisseria gonorrhoeae*, *Human papillomavirus*, *Herpes simplex virus*, *Candida albicans* and *Ureaplasma parvum*, but the *23S rRNA* assay cross-reacted with *Mycoplasma pneumoniae*. Compared with sequencing results, the sensitivity of *23S rRNA* was 100% (95% CI; 93.3 -100), the specificity was 94.3% (95% CI; 79.4 - 99.0), the overall consistency was 98% (95% CI; 92.5 - 99.7) and *kappa* value was 0.96 (*P* < 0.001); the sensitivity of *parC* was 100% (95% CI; 93.4 - 100), the specificity was 89.7% (95% CI; 71.5 - 97.3) and the overall consistency was 96.9% (95% CI; 90.7 - 99.2) with a *kappa* value of 0.92 (*P* < 0.001).

**Conclusions:**

The results of this sensitive and rapid alternative for identifying resistant genotypes of *Mycoplasma genitalium* are intuitive and easy to interpret, especially for mixed MG populations. Although the relevant *23S rRNA* primers need further adjustment, this reliable method would provide an effective diagnostic tool for the selection of antibiotics in clinical practice.

## Introduction

*Mycoplasma genitalium* (MG) is one of the most common sexually transmitted pathogens in the clinic. MG infection is correlated with non-gonococcal urethritis, vaginitis, pelvic inflammatory disease and tubal infertility (Lis et al., 2015). It is also a suspected cause of preterm birth, reactive arthritis and proctitis (Daley et al., 2014; Gnanadurai and Fifer, 2020). In recent years, drug-resistant mutation rates to first-line (macrolides) and second-line (fluoroquinolones) treatment regimens have increased rapidly worldwide (Machalek et al., 2020), with significant regional variations. Individualized treatment based on macrolide resistance detection has been recommended in the United States (Workowski et al., 2021), the United Kingdom (Soni et al., 2019), Australia (The Australian Government Department of Health, 2021) and other regions (Jensen et al., 2022). Recent studies have shown that macrolide resistance is strongly associated with mutations at 2058 and 2059 (*E. coli* numbers) positions of the *23S rRNA*, whereas fluoroquinolone resistance is mainly associated with amino acid substitutions at S83 and D87 (*M. genitalium* numbers) in the quinolone resistance determining region (QRDR) of *parC*. Amino acid substitutions in the QRDR of *gyrA* may have a synergistic effect (Bissessor et al., 2015; Nijhuis et al., 2015; Fernandez-Huerta et al., 2020; Murray et al., 2020; De Baetselier et al., 2021; De Baetselier et al., 2022). Sequencing is widely used as the “gold standard” for mutation detection in clinical research. However, its value in clinical applications is limited by the complexity of the processes and the lack of professional instrumentation in some laboratories. Although real-time fluorescent PCR-based commercial kits or self-constructed methods are available and evaluated, they usually have limitations, such as detecting only one single target, either *23S rRNA* or *parC*, lack of accurate genotype identification and unpublished primer and probe sets (Wold et al., 2015; Thellin et al., 2019; Le Roy et al., 2020; Gullsby et al., 2021; Shedko et al., 2021). To provide a flexible and rapid guide to clinical antibiotic therapy, this study aims to establish a nested real-time PCR assay for key sites associated with macrolide and fluoroquinolone resistance, including mutations at *23S rRNA* A2058, A2059 and *parC* S83, D87 positions.

## Materials and methods

### Sample collection

From April 2022 to March 2023, 105 MG-positive samples were used as validation samples for nested real-time PCR experiments to evaluate laboratory performance, such as concordance rate with Sanger sequencing method, limit of detection (LoD), and so on. Of these samples, 17 were male urogenital swabs and 88 were female urogenital swabs; 87 cases were from pre-treatment samples and 18 cases were from test-of-cure (TOC) samples. Each three specimens with different common urogenital pathogens and *Mycoplasma pneumoniae* have been used for cross-reactivity validation, respectively. The study was approved by the Medical Ethics Committee of the Third Affiliated Hospital of Guangzhou Medical University (No.2022044).

### Nested real-time PCR assay

MG was detected by real-time simultaneous amplification and testing (SAT) (Rendu Biotechnology Co., Shanghai, China) with a LoD of 1000 copies/reaction. Nucleic acid was extracted from 300μL eluent of positive samples using the Smart Lab Automatic Nucleic Acid Extractor (Advanced Nanotech Co., Taiwan, China) and DNA/RNA Extraction Kit (Health Gene Technologies Co., Ningbo, China). 50μL of nucleic acid samples are stored at -80°C until use.

Step 1: The first round of PCR experiments was performed according to the instructions of the Platinum SuperFi II PCR Master Mix Kit (CAT# 12368010, Thermo Fisher, USA) with a total reaction volume of 50 μL: 25 μL of 2 × Platinum SuperFi II PCR Master Mix, 2.5 μL primers (10 μM), 5 μL nucleic acid sample and 15 μL water. The primers used as follows: *23S rRNA* 5’- CAACTCTATGCCAAACCGTAAG-3’ and 5’-GTC AAACTGCCCACCTAACAC-3’, *parC* 5’-AAACCAGTACAAAGACGGATCT-3’ and 5’-GAGGTTAGGCAGTAAGGTTGG-3’.

The reaction was performed on a Veriti PCR instrument (Applied Biosystems, USA) under the following PCR conditions: 98°C, 30 seconds, 1 cycle; 98°C, 10 seconds, 60°C, 10 seconds, 72°C, 30 seconds, 35 cycles; 72°C, 5 minutes and 4°C hold.

Step 2: The PCR product of Step1 was diluted 1:1000, and real-time PCR was performed according to the manual of the FastFire qPCR PreMix (Probe) kit (CAT# FP208, Tiangen Biotechnology Co., Beijing, China), with a total reaction volume of 20 μL, including 10 μL of 2 × qPCR Mix, 0.4 μL primers (10 μM), 0.2 μL probes (10 μM), 2 μL diluted Step1 PCR product, and 7 μL water. A Cobas z480 fluorescence PCR instrument (Roche Diagnostics, Germany) was used for detection, with the following reaction conditions: 95°C, 5 minutes, 1 cycle; 95°C, 10 seconds, 70°C, 30 seconds (FAM channel acquisition), 25 cycles; 37°C, 30 seconds, 1 cycle. All primers and probes above were designed by Primer Premier software (Version 6, Premier Biosoft Inc., CA), then were synthesized by Guangzhou IGE Biotechnology Co. Primer and probe sequences used in fluorescent PCR experiments are shown in **Table 1**. Seven probes were used to detect mutations at A2058 and A2059 positions of *23S rRNA*, one probe to detect A2060 of *23S rRNA* was used as a negative control, four probes were used to detect mutations at S83 amino acid site of *parC*, and four probes were used to detect mutations at D87 amino acid site of *parC*.

**Table 1:** Primers and probes for Step2: Real-time fluorescence PCR experiments.

Detection Site	Primers and Probes	Sequence (5’-3’)	Genotype
*23S rRNA* A2058、 A2059	23S-1991F 23S-2137R	CCATCTCTTGACTGTCTCGGCTAT CCTACCTATTCTCTACATGGTGGTGTT	
	S1	FAM- ACGG **AA** AGACCCCGT -MGB	Wild type
	S2	FAM- ACGGA **C** AGACCCCGT -MGB	A2059C
	S3	FAM- ACGGA **G** AGACCCCGT -MGB	A2059G
	S4	FAM- ACGGA **T** AGACCCCGT -MGB	A2059T
	S5	FAM- ACGG **C** AAGACCCCGT -MGB	A2058C
	S6	FAM- ACGG **G** AAGACCCCGT -MGB	A2058G
	S7	FAM- ACGG **T** AAGACCCCGT -MGB	A2058T
	S8	FAM- ACGGAA **G** GACCCCGT -MGB	A2060G (NC)
*ParC* S83	ParC-213F ParC-295R	GGAGATCATGGGGAAATACCA TGTTCTTTCAGCTTTGGGACA	
	P1	FAM- CATGGTGAT **AG** TTCC -MGB	Wild type
	P2	FAM- CATGGTGAT **C** GTTCC -MGB	A247C (S83R)
	P3	FAM- CATGGTGATA **T** TTCC -MGB	G248T (S83I)
	P4	FAM- CATGGTGATA **A** TTCC -MGB	G248A (S83N)
*ParC* D87	ParC-213F ParC-295R	GGAGATCATGGGGAAATACCA TGTTCTTTCAGCTTTGGGACA	
	P5	FAM- CCATTTAT **GA** TGCAATTATC -MGB	Wild type
	P6	FAM- CCATTTAT **A** ATGCAATTATC -MGB	G259A (D87N)
	P7	FAM- CCATTTAT **T** ATGCAATTATC -MGB	G259T (D87Y)
	P8	FAM- CCATTTATG **G** TGCAATTATC -MGB	A260G (D87G)

Mutation sites are highlighted with bold and underline.

In order to check the amplification efficiency of the fluorescent PCR phase, wild-type plasmids were diluted in a ten-fold gradient, followed by detecting with S1 and P1 primer/probe set. The primer/probe efficiency was calculated automatically by Roche z480 instruments based on the standard curves from three independent experiments.

### Limit of detection

Twelve pGSI plasmids containing different mutant fragments (*23S rRNA* A2058C, A2058G, A2058T, A2059C, A2059G, A2059T; *parC* A247C (S83R), G248T (S83I), G248A (S83N), G259A (D87N), G259T (D87Y), A260G (D87G) and the pGSI plasmid containing the wild-type fragment were used to validate the LoD of the overall experimental protocols. Different plasmids were diluted to 100000, 10000, 1000, 500, 200, 100 and 50 copies/reaction and the experiments were repeated at least ten times. The lowest concentration with 100% amplification is the LoD of each genotype and the LoD of the whole experiment is the highest LoD of all genotypes.

### Cross reaction

Twenty-four urogenital swab specimens and three pharyngeal swab specimens of *Mycoplasma pneumoniae* were confirmed for the presence of different pathogens using National Medical Products Administration (NMPA)-approved reagents from Liferiver BioTech Co., including *Ureaplasma urealyticum*, *Mycoplasma hominis*, *Chlamydia trachomatis*, *Neisseria gonorrhoeae*, *Human papillomavirus*, *Herpes simplex virus*, *Candida albicans*, *Ureaplasma parvum* and *Mycoplasma pneumoniae*. All these samples were MG-negative and were used to verify the cross reaction of the nested real-time PCR assay with other pathogens.

### Accuracy

105 MG*-*positive samples were detected by the evaluated method and Sanger sequencing respectively, and the consistency of the results was assessed.

All the Step1 PCR products of MG-positive samples were subjected to 1% agarose gel electrophoresis before sequencing to analyze their specificity, and then sequencing experiments were performed by Guangzhou IGE Biotechnology Co. Step1 PCR products were sequenced in both directions, then were analyzed by Chromas software and compared with *Mycoplasma genitalium* G37 strain genome sequence (GenBank NC _ 000908). The *23S rRNA* mutation sites are represented by the *E. coli* numbers. The *parC* mutations and amino acid sites are represented by the *Mycoplasma genitalium* numbers. Data were analyzed using SPSS software, version 19.0 (IBM Co., Armonk, NY, USA). All data were expressed and analyzed as crosstabs, the Wilson test with a correction for continuity was used to calculated the 95% confidence interval (CI) of a proportion.

## Results

### Presentation of nested real-time PCR results

**Figure 1:**
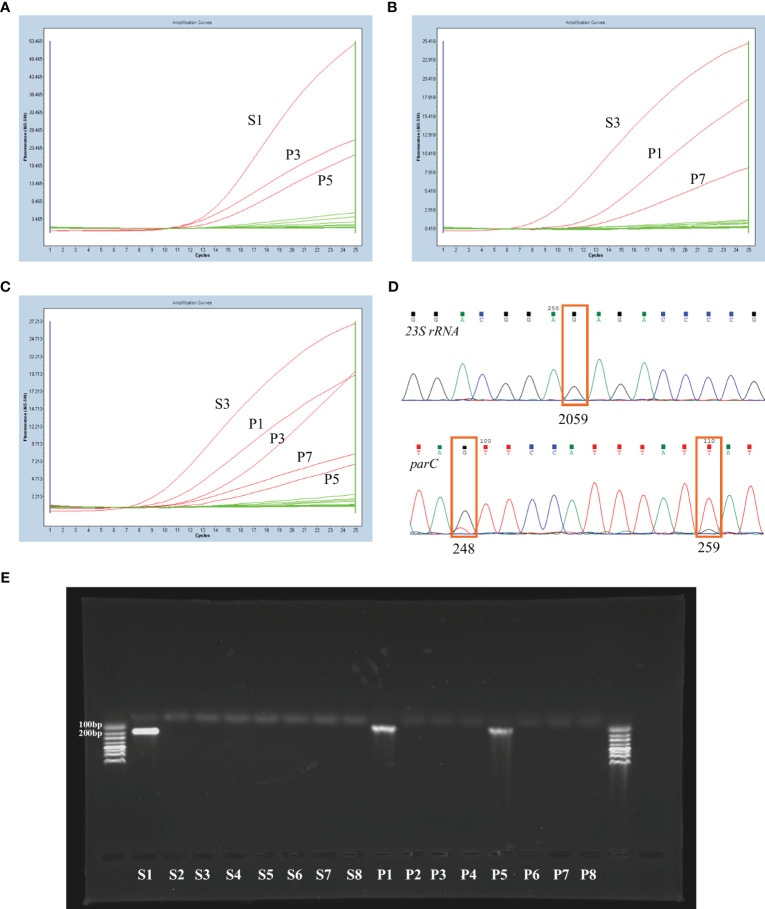
Examples of the nested RT-PCR experiment and corresponding confirmation experiment results. **(A)** The nested RT-PCR results of a MG-positive sample with *23S rRNA* WT and *parC* G248T (S83I) genotypes. **(B)** The nested RT-PCR results of a MG-positive sample with *23S rRNA* A2059G and *parC* G259T (D87Y) genotypes. The nested RT-PCR **(C)** and corresponding sequencing results **(D)** of a sample with mixed MG populations. **(E)** The agarose gel electrophoresis results of the real-time fluorescent PCR products (step 2) from a wild-type sample.

For each sample, 16 fluorescent PCR amplification reactions were performed simultaneously (including a negative control). The *23S rRNA* and *parC* genotypes of each sample were assessed comprehensively by combining the detection curves of seven *23S rRNA* probes and eight *parC* probes. The fluorescent PCR detection results of the three independent samples are shown in **Figure 1**. If the sample contain a single MG population, three amplification curves will appear, respectively for *23S rRNA*, *parC* S83 and *parC* D87 regions, as shown in **Figures 1A** , **B**. The reactions with the S1, P3 and P5 probes showed an S-shaped amplification curve in the sample shown in **Figure 1A**, and the corresponding genotypes were *23S rRNA* WT and *parC* G248T (S83I). The reactions with the S3, P1 and P7 probes showed an S-shaped amplification curve respectively in another sample shown in **Figure 1B**, and the corresponding genotypes were *23S rRNA* A2059G and *parC* G259T (D87Y).

In fact, mixed MG populations may be present in individual samples. **Figures 1C, D** showed the nested real-time PCR and sequencing results of one sample containing mixed mutants. The reactions of nested real-time PCR with probes S3, P1, P3, P7 and P5 exhibited an S-shaped amplification curve, and the corresponding genotypes were *23S rRNA* A2059G, *parC* G248T (S83I)/G259T (D87Y) or G248T (S83I)/G259T (D87Y)/WT. These mixed MG populations could also be distinguished by the sequencing results.

To demonstrate the specificity of this method, 1% agarose gel electrophoresis was performed with the real-time PCR products (Step 2) from a wild-type sample. The results showed that there were bands in the S1, P1 and P5 lanes (**Figure 1E**), with the S1 fragment 147 bp in length and the P1 and P5 fragment 83 bp in length, as expected.

### Performance validation of the nested real-time PCR assay

In the real-time fluorescence PCR stage (Step2), the S1 primer/probe set efficiency was 1.900 ± 0.021, and the P1 primer/probe set efficiency was 1.838 ± 0.044.

Plasmids with different concentrations were detected ten times with the corresponding probes respectively. At a concentration of 200 copies/reaction, all *23S rRNA* reaction systems can be amplified, whereas at a concentration of 500 copies/reaction, all *parC* reaction systems can be amplified. In summary, the LoD of the nested real-time PCR was 500 copies/reaction.

The assay didn’t cross-react with common urogenital pathogens such as *Ureaplasma urealyticum*, *Mycoplasma hominis*, *Chlamydia trachomatis*, *Neisseria gonorrhoeae*, *Human papillomavirus*, *Herpes simplex virus*, *Candida albicans* and *Ureaplasma parvum* (**Figures S1A–H**). There were no S-shaped amplification curves for any of the assay systems. However, the *23S rRNA* assay cross-reacted with *Mycoplasma pneumoniae* (**Figure S1I**).

The detection rate of the nested real-time PCR method was 100% (105/105). *23S rRNA* sequencing failed in 2 samples and *parC* sequencing failed in 7 samples. A comparison of the nested real-time PCR and Sanger sequencing methods is shown in **Table 2**. Most of the results were consistent. However, in 5 samples, the results of the two methods were not consistent, including 2 *23S rRNA* WT by sequencing but WT/A2059G by the nested real-time PCR, 1 *parC* WT by sequencing but WT/G259A (D87N) by the nested real-time PCR, and 2 *parC* WT by sequencing but WT/G248T (S83I) by the nested real-time PCR. The evaluated method showed that there were mixed MG populations in the sample, but the sequencing results showed that it was unable to distinguish the low peak at the mutation site from background noise on the sequencing map.

**Table 2:** Comparison of single nucleotide polymorphism (SNP) detection for the nested RT-PCR assay compared to Sanger sequencing.

		Sanger sequencing		
		Mutant	Wild-type	Total
**Nested RT-PCR** *23S rRNA*	Mutant	68^a^	2^b^	70
	Wild-type	0	33	33
	Total	68	35	
**Nested RT-PCR** *parC*	Mutant	69^c^	3^d^	72
	Wild-type	0	26	26
	Total	69	29	

a 1 A2058C, 7 A2058G, 3 A2058T, 0 A2059C, 50 A2059G, 0 A2059T, 6 WT/A2059G, 1 WT/A2058G mutations were included.

b Determined to be WT by sequencing, but WT/A2059G by nested RT-PCR.

c 2 A247C (S83R), 43 G248T (S83I), 3 G248A (S83N), 4 G259A (D87N), 12 G259T (D87Y), 0 A260G (D87G), 1 G248T (S83I)/G259T (D87Y) or G248T (S83I)/G259T (D87Y)/WT, 1 WT/G259A (D87N), 3 WT/G248T (S83I) were included.

d 1 WT by sequencing, but WT/G259A (D87N) by nested RT-PCR; 2 WT by sequencing, but WT/G248T (S83I) by nested RT-PCR.

The sensitivity and specificity of *23S rRNA* were 100% (95% CI; 93.3 - 100) and 94.3% (95% CI; 79.4 - 99.0), and the consistency was 98% (95% CI; 92.5 - 99.7) with a *kappa* value of 0.96 (*P* < 0.001). The sensitivity for *parC* was 100% (95% CI; 93.4 - 100), the specificity was 89.7% (95% CI; 71.5 - 97.3) and the consistency was 96.9% (95% CI; 90.7 - 99.2). The *kappa* value was 0.92 (*P* < 0.001).

## Discussion

The guideline-recommended accompanying detection of macrolide resistance-associated *23S rRNA* mutations in MG has been widely used for individualized treatment of MG infection in some areas, including the UK, USA and Australia. However, there is still debate on how to predict the therapeutic response to fluoroquinolones by MG genotypes, as studies have not found a strong concordance between them (Manhart and Jensen, 2020; Sweeney et al., 2022). In China, our previous study showed that the prevalence rate of *23S rRNA* mutations at A2058 and A2059 positions in MG samples was 64%, and the amino acid substitution rate of *parC* S83 and D87 was 67.5% (Li et al., 2023). In such areas with high mutation rates, it is particularly important to try to estimate both macrolide and fluoroquinolone resistance.

In recent years, in addition to the sequencing methods, researchers have developed a variety of methods to detect *23S rRNA* or *parC* resistance mutation sites in *Mycoplasma genitalium*, including high-resolution melting curve PCR (HRM-PCR) (Twin et al., 2012; Li et al., 2022), amplification refractory mutation system PCR (ARMS-PCR) (Wold et al., 2015), and commercial kits based on multiplex fluorescent PCR methods (Le Roy et al., 2021; Gardette et al., 2022).

Compared to sequencing, the established nested real-time PCR method could be performed in most clinical laboratories (fluorescent PCR instrument is also the main equipment for SARS-CoV-2 detection), but this method takes less time with high sensitivity and specificity. In addition, the result of this method is more intuitive than HRM technologies. Compared with the existing real-time fluorescent PCR-based commercial kits, this assay can detect two resistance-related genes simultaneously and can be expanded by designing probes according to the local genotypes. However, the assay has some limitations. Firstly, cross-reaction with *Mycoplasma pneumoniae* may occur when *23S rRNA* mutations are detected, so the relevant primers need further adjustment. Secondly, each sample requires sixteen parallel reactions to be performed. To enhance detection throughput and decrease expenses, it is feasible to apply distinct fluorescence markers for labelling probes on the same locus. Thus, mutations at the same locus can be detected in combination. In short, this research shares a practical tool for monitoring macrolide and fluoroquinolone resistance of *Mycoplasma genitalium* in urogenital swab specimens.

## Data availability statement

The original contributions presented in the study are included in the article/**Supplementary Material**. Further inquiries can be directed to the corresponding authors.

## Ethics statement

The studies involving humans were approved by the Medical Ethics Committee of the Third Affiliated Hospital of Guangzhou Medical University. The studies were conducted in accordance with the local legislation and institutional requirements. The participants provided their written informed consent to participate in this study.

## Author contributions

LL: Funding acquisition, Writing – original draft, Writing – review & editing, Project administration. WH: Methodology, Validation, Writing – original draft. YY: Methodology, Validation, Writing – original draft. JL: Data curation, Formal Analysis, Writing – original draft. XF: Data curation, Formal Analysis, Writing – original draft. XP: Writing – original draft, Writing – review & editing, Supervision.

## Supplementary material

Supplementary Figure 1 The cross reaction of the nested RT-PCR assay with other common pathogens.
