# Supplementary figures and images for: Detection of macrolide and fluoroquinolone resistance-associated 23S rRNA and parC mutations in Mycoplasma genitalium by nested real-time PCR
Source: Front Cell Infect Microbiol. 2023 Oct 20;13:1271392. doi: 10.3389/fcimb.2023.1271392 (PMC10623348; doi:10.3389/fcimb.2023.1271392)

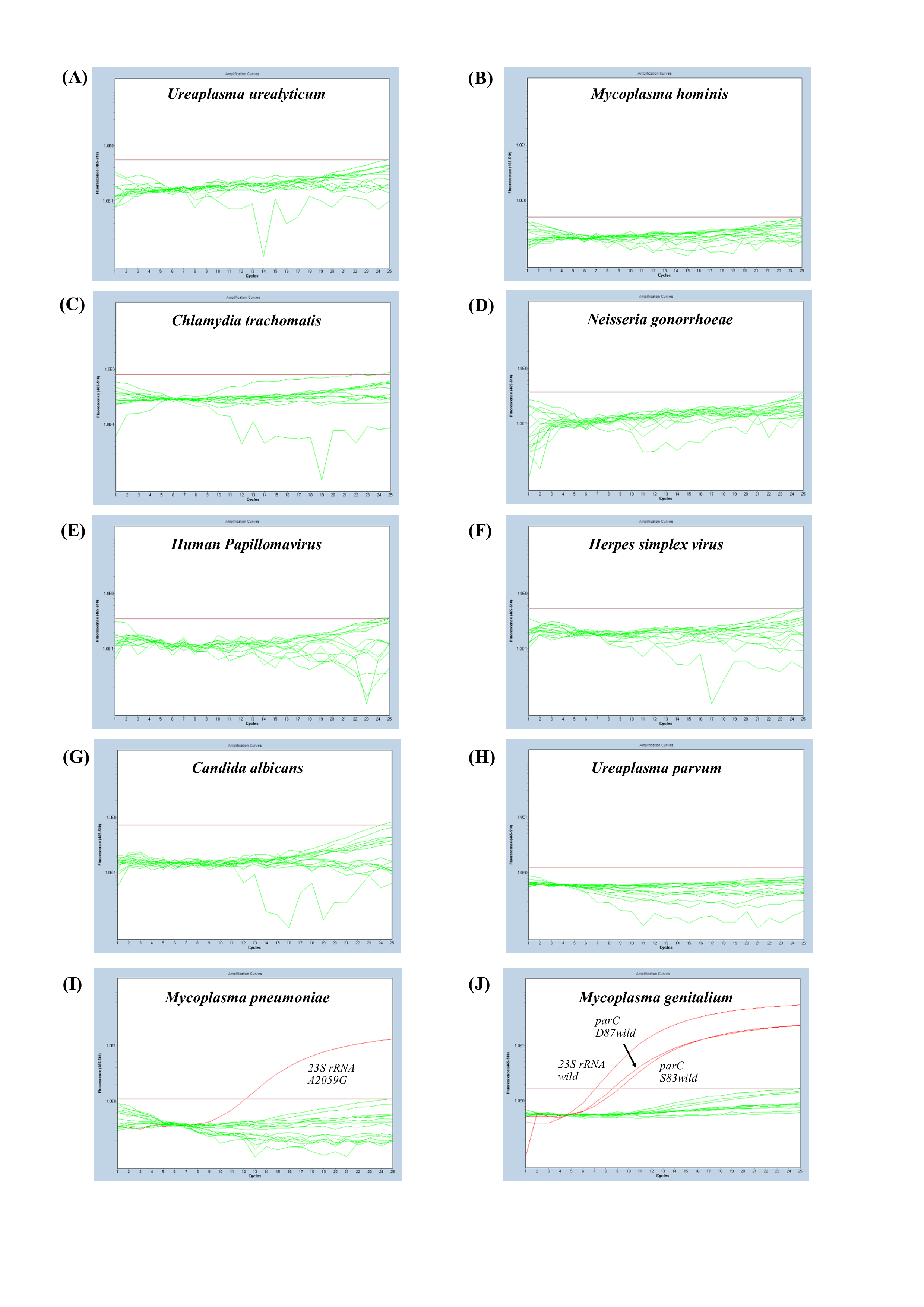

Supplement: Supplementary Figure 1 — The cross reaction of the nested RT-PCR assay with other common pathogens. [file Image_1.tif]
